# Population genomics and morphological features underlying the adaptive evolution of the eastern honey bee (*Apis cerana*)

**DOI:** 10.1186/s12864-019-6246-4

**Published:** 2019-11-15

**Authors:** Li Yancan, Chao Tianle, Fan Yunhan, Lou Delong, Wang Guizhi

**Affiliations:** 1 Shandong Provincial Key Laboratory of Animal Biotechnology and Disease Control and Prevention, Shandong Agricultural University, 61 Daizong Street, Tai’an, 271018 Shandong Province People’s Republic of China; 2 Shandong Apiculture Breeding of Improved Varieties and Extension Center, 186 Wuma Street, Tai’an, 271000 Shandong Province People’s Republic of China

**Keywords:** *Apis cerana*, *Apis mellifera*, Genomics, Population genetics, Genetic differentiation, Population structure, Standard morphometrics

## Abstract

**Background:**

The adaptation of organisms to changing environments is self-evident, with the adaptive evolution of organisms to environmental changes being a fundamental problem in evolutionary biology. Bees can pollinate in various environments and climates and play important roles in maintaining the ecological balance of the earth.

**Results:**

We performed an analysis of 462 *Apis cerana* (*A. cerana*) specimens from 31 populations in 11 regions and obtained 39 representative morphological features. We selected 8 *A. cerana* samples from each population and performed 2b-RAD simplified genome sequencing. A total of 11,506 high-quality single nucleotide polymorphism (SNP) loci were obtained. For these SNPs, the minor allele frequency (MAF) was > 1%, the average number of unique labels for each sample was 49,055, and the average depth was 72.61x. The ratios of the unique labels of all samples were 64.27–86.33%.

**Conclusions:**

Using 39 morphological characteristics as the data set, we proposed a method for the rapid classification of *A. cerana*. Using genomics to assess population structure and genetic diversity, we found that *A. cerana* has a large genetic difference at the ecotype level. A comparison of *A. cerana* in North China revealed that some physical obstacles, especially the overurbanization of the plains, have isolated the populations of this species. We identified several migration events in North China and Central China. By comparing the differences in the environmental changes in different regions, we found that *A. cerana* has strong potential for climate change and provides a theoretical basis for investigating and protecting *A. cerana*.

## Background

Adaptation to a changing environment is a fundamental problem in evolutionary biology. Understanding the impact of the environment on biological genetic diversity can not only clarify the course of evolution but can also provide information on how to protect the ecological environment [1, 2]. Bees have unique ecological value, and one-third of human food is derived from insect pollination, which is critical for maintaining the dynamics of the ecosystem. Bees are important mediators of pollination, bringing ecological value of more than 200 billion US dollars per year [3, 4].

*Apis* contains 10 different species, most of which are distributed throughout Asia [5]. *A. cerana* is an Asian honeybee species with a wide geographical distribution. Previously, *A. cerana* was divided into five or six local categories based on a comprehensive study of mitochondrial DNA and microsatellite DNA, and the *A. cerana* species in China belonged to a subspecies [6, 7]. *A. cerana* was defined as one of the most important pollinators in China, domesticating the region for more than 2000 years [8]. The various climate types and complex terrains in China have promoted bee differentiation. Previous studies on the differentiation of *A. cerana* in China have classified *A. cerana* into different ecological types only by morphometric measurements. However, the results were variable due to different measurement indicators [9, 10]. It is generally accepted that there are nine ecotypes of *A. cerana* in China, including Hainan, the Yunnan-Guizhou Plateau, Tibet, Aba, Changbai Mountain, southern Yunnan, North China, South China, and Central China [11]. However, these results were mainly based on the classification method of *A. mellifera*, and the conclusions were obtained from no more than 12 traits; therefore, the unique features of *A. cerana* were likely missing from the analysis [12]. A subsequent study used the mitochondrial tRNALeu-COII region to classify *Apis mellifera* (*A. mellifera*) [6, 13–17]. As more investigators began to study the genome, our understanding of *A. cerana* continued to improve until 2015, when the reference genome was published [18]. Recently, Chen et al. conducted a comprehensive genomics study of *A. cerana* in most parts of China, which paralleled the simple hierarchical division and proposed that physical barriers (such as islands and mountains), rather than physical distances, lead to population differentiation. In other words, these barriers are the main obstacles leading to population exchange [19]. Other studies have reported that *A. cerana*, even in the same region in China, can be highly variable [17, 20–23], which has prompted us to study this species.

*A. cerana* uses small-scale nectar plants effectively. This species also has a strong harvesting ability, a long honey-collecting period and adaptability, strong disease resistance, and low consumption of feed, which are highly suitable for changing landscapes. The number of *A. cerana* has fallen sharply in recent decades [24], with climate change being one of the main threats to this species [25]. On the one hand, *A. cerana* has unique economic and ecological values in agriculture in China. In recent years, due to the continuous increase of China’s population, *A. cerana* has helped to maintain a balance between demand and production by contributing to high yields of grain. On the other hand, *A. cerana* is at the center of research in evolutionary biology, which attempts to understand how the population responds to climate change or environmental insults, thereby providing basic knowledge of the evolution and adaptation of *A. cerana*.

In this study, we collected samples from regions of different topography, environment, and climate and carried out a comprehensive morphometric determination, with the *A. cerana* genome [18] serving as the basic resource. The accuracy of the division of *A. cerana* can also be used to determine the genetic variation of the adaptive and economic shape, thereby providing an in-depth understanding of the genetic structure of *A. cerana* as a whole. We conducted a more comprehensive measurement of North China and analyzed the effects of topographical and environmental changes, including human activities, by combining morphological characteristics and genomic differences. We identified genetic variations, proposed a rapid classification method, explored the population structure and factors affecting differentiation, and screened the *A. cerana* genes that adapted to the local environment, which provides evidence of genetic diversity for future research investigating *A. cerana.*

## Results

### Population structure

The morphological measurements of the collected *A. cerana* samples included: (1) Bee right front wing: length of bee right forewing cubital vein a (a), length of bee right forewing cubital vein b (b), width of bee right forewing (FB), length of bee right forewing (FL), angle A4, B4, D7, E9, G18, J10, J16, K19, L13, N23, O26 of bee forewing (A4, B4, D7, E9, G18, J10, J16, K19, L13, N23, O26), bee index number of cubital vein (Ci); (2) Bee right hind wing: Bee branches midrib (NT), Number of bee hindwing hamule (Nh); (3) Bee tergum: bee length of tergum 3,4 (T3, T4), bee width of tomentum on tergum 5 (T5); (4) Bee sternum: (bee length of sternum 7 (L7), bee width of sternum 7 (T7), bee length of sternum 4 (S4), bee distance of bee wax mirror on sternum 4 (WD), bee length of bee wax mirror on sternum 4 (WL), bee width of bee wax mirror on sternum 4 (WT); (5) Bee hind leg (length of femur of bee hind leg (Fe), length of bee hind leg basitarsus (ML), width of bee hind leg basitarsus (MT), length of tibia of bee hind leg (Ti); (6) Color of the bee: bee pigment of scutellum B,K area (B, K), bee pigment of tergum 2,3,4 (P2, P3, P4), bee pigment of clypeus (Pc), bee pigment of labrum (PL), and bee pigment of scutellum Sc area (Sc) (Additional file 2: Table S1). The abbreviations corresponding to these 39 traits and other names are located in Additional file 3: Table S2. The results showed significant differences between the different regions (*P* < 0.01).

**Fig. 1:**
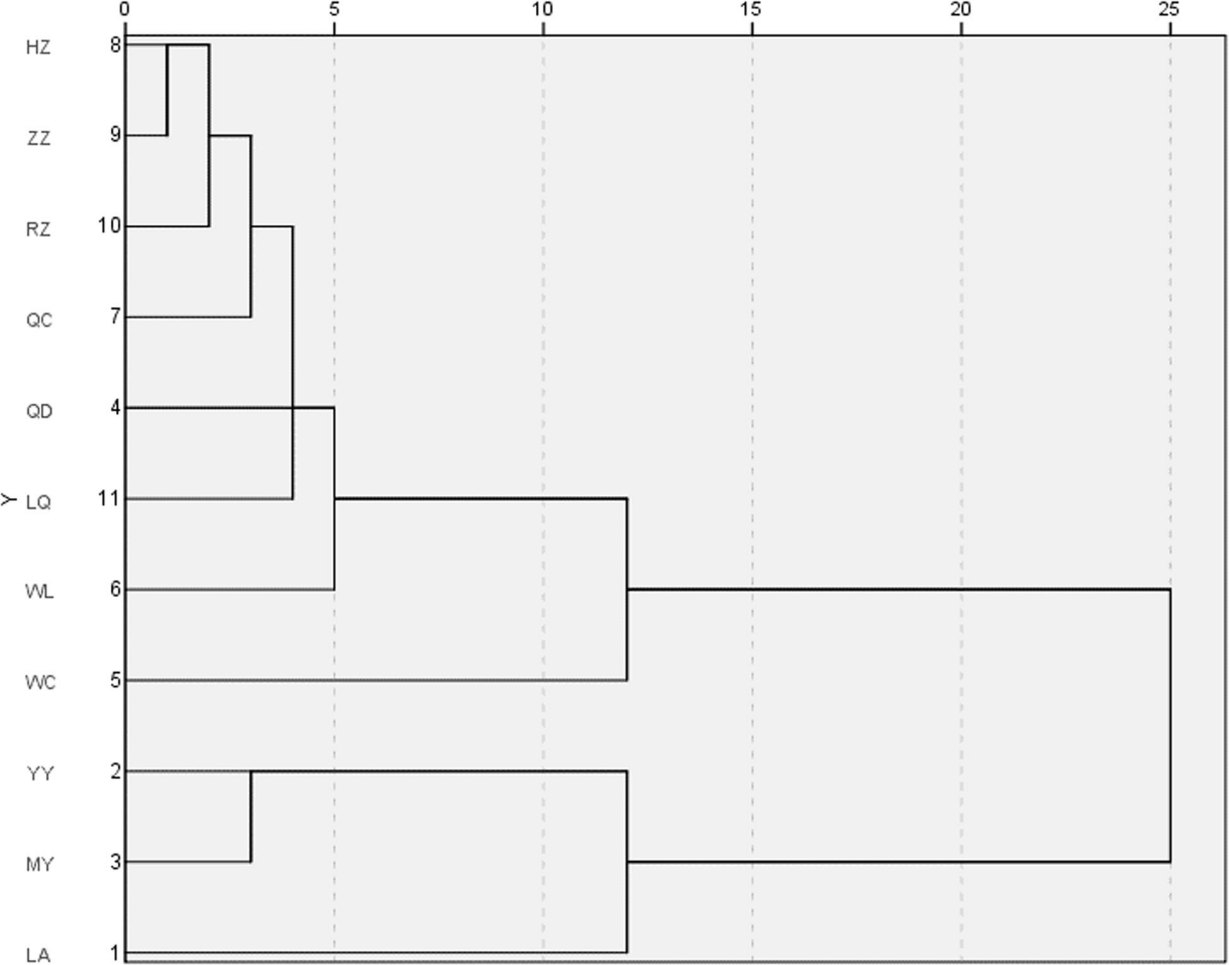
Cluster analysis based on 39 morphological indicators of *A. cerana* from 11 regions

We divided 462 *A. cerana* from 31 populations collected from 11 regions in China (Mengyin (MY), Yiyuan (YY), Rizhao (RZ), Qingdao (QD), Linqu (LQ) and Zaozhuang (ZZ) Chongqing (WL), Sichuan (QC), Anhui (LA), Shanxi (HZ) and Hainan (WC)) and used the average of the morphological data of *A. cerana* from the 11 regions for the cluster analysis. Figure 1 shows the *A. cerana* interregional relationships. At a threshold of 12, *A. cerana* from the 11 regions was divided into 4 groups, in which YY and MY were grouped together, LA and WC were in separate groups, and the remaining seven regions were clustered into one large group. According to the Chinese recognized ecotypes of *A. cerana*, YY, MY, LA, QD, ZZ, RZ, and LQ were from the North China ecological type; WC was sampled from the Hainan ecological type; and HZ, WL, and QC were from the Central China ecological type [11] (National Commission for Animal Genetic Resources 2011). Although ZZ, RZ, QD, and LQ were from North China, they were clustered into a group associated with Central China ecological type according to our measurements.

**Fig. 2:**
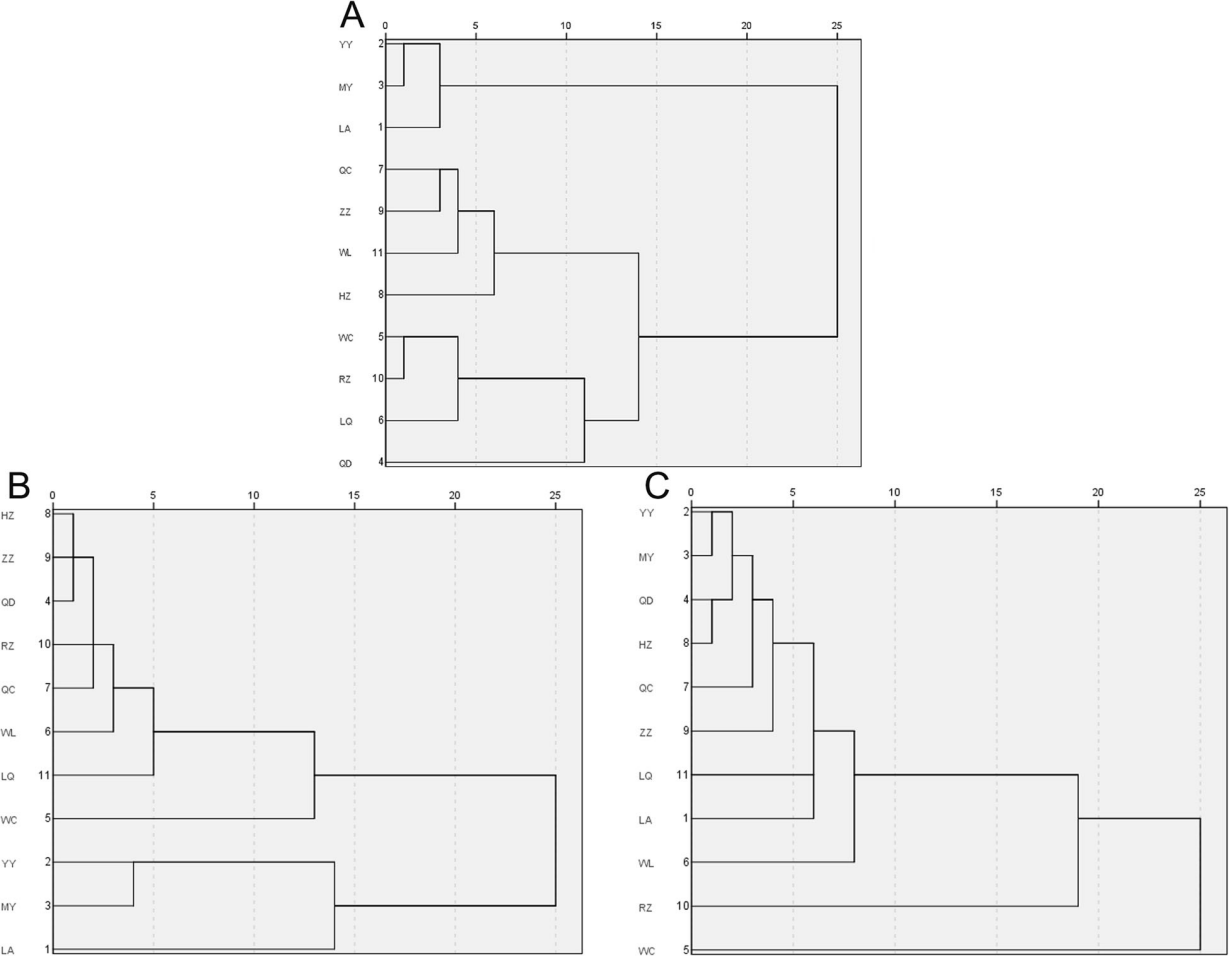
**a** Cluster analysis of color using eight indicators, including B, K, P2, P3, P4, Pc, PL, and Sc. **b** Cluster analysis of angle of right forewings using 12 indicators, including A4, B4, D7, E9, G18, J10, J16, K19, L13, N23, Nh, and O26. **c** Cluster analysis of body size traits using 19 indicators, including a, b, Ci, FB, Fe, FL, L7, ML, MT, NT, S4, T3, T4, T5, T7, Ti, WD, WL, and WT

We clustered the 39 traits into three categories—body size, color type, and angle of right forewing—for cluster analysis (Fig. 2). We found differences in the clustering results, and different trait categories revealed different characteristics for the bees in each area. According to the color classification, we divided *A. cerana* from the 11 regions into three groups as follows: YY, MY, and LA formed the first group; ZZ, LQ, QC, and HZ fell into the second group; and WC, RZ, WL, and QD formed the third group. This finding was notably different from the results we had used to classify all the trait data. In particular, RZ, LQ, QD, and WC were relatively close in the four regions of the third group. We found that these regions were located in coastal areas, which seemed to be related to the color of the bees. According to the results of body size clustering, there was a significant difference in the size of the Hainan Bee (WC) compared to other bees, whereas clustering according to the angle of right forewing showed no difference between the 12 indicators of wing geometry and the total 39 traits.

**Fig. 3:**
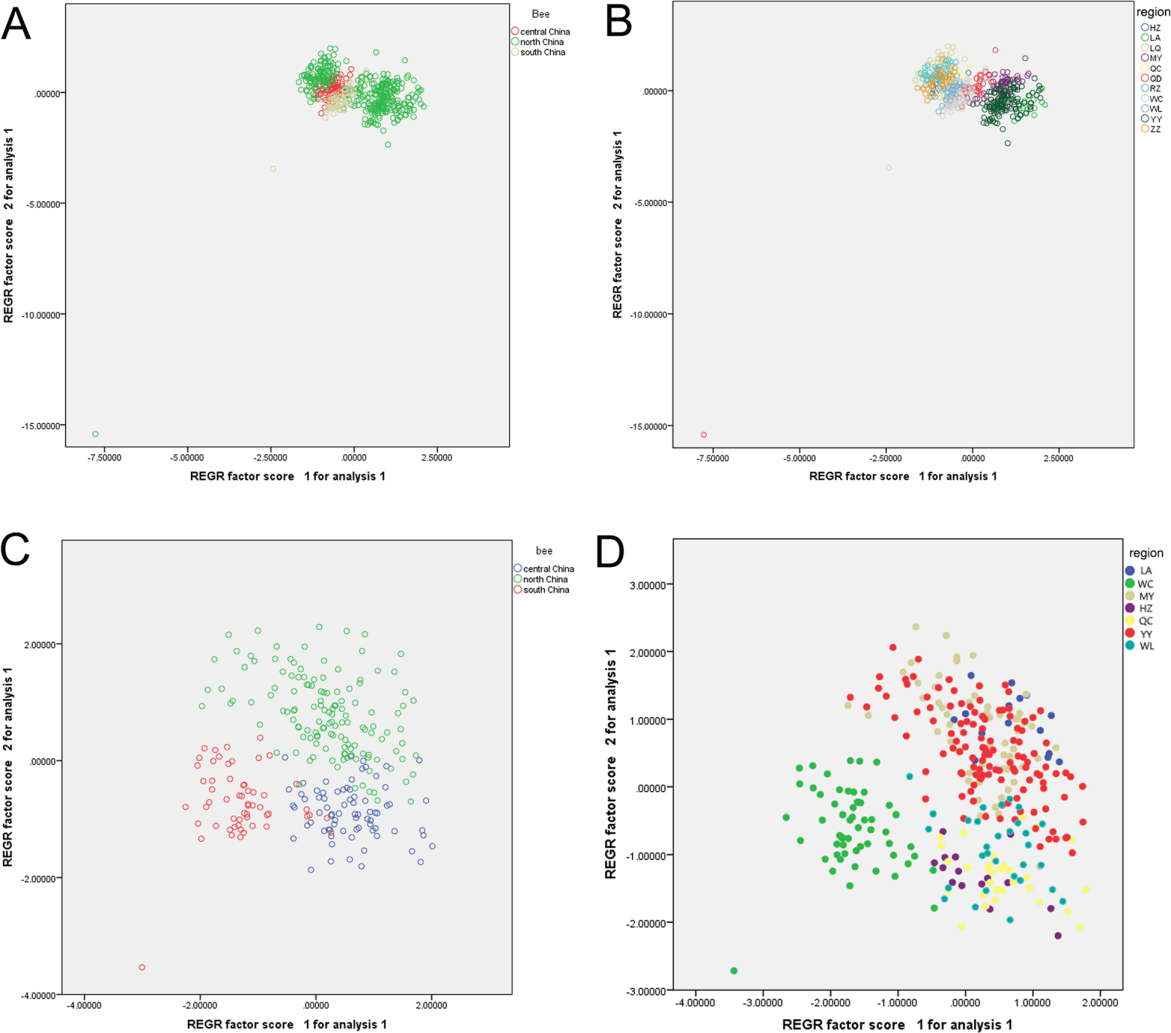
Principal component analysis of the population structure in different regions of *A. cerana*. **a** Scatter plot of the main component 1 to 2 (PCA1 vs PCA2) from North China, Central China, and Hainan. **b** Scatter plot of the main component 1 to 2 (PCA1 vs. PCA2) from the 11 regions of LA, WC, LQ, MY, QD, RZ, HZ, QC, YY, ZZ, and WL (**c**) After removing the regions of RZ, ZZ, LQ, and QD, the RZ, ZZ, LQ, and QD regions were removed according to the sampling region in the scatter plot (**d**) of the main factor 1 and main factor 2. The main factor 1 and main factor 2 were passed according to the sampling region in the scatter plot

We performed principal component analysis using individual values and selected the first two factors with cumulative variability values of 57.4%. To facilitate the observations, we divided the collected bees according to the *A. cerana* ecological type of the sampling area. The northern ecological type was divided into two regions, the larger region and the smaller region that overlapped with the central ecological type. The central and south ecological types were nearby, but the division was clear (4A). When the bees were divided according to the sampling region, the bees in the 11 regions grouped together more obviously. The distribution pattern was consistent with the morphometric measurement of 39 traits. RZ, ZZ, LQ, and QD, which belong to North China, are close to central China (Fig. 1, 3a). We attempted to remove them and observe them again. The regions are clearly divided by the scatter plot of principal factor 1 and principal factor 2 (Fig. 3c, Fig. 3d) and divided *A. cerana* into three categories. In general, the population distance is consistent with the geographical distance, but there is a contradiction between the North China region and its corresponding ecological type.

### Population genetic structure analysis

#### Population structure

**Fig. 4:**
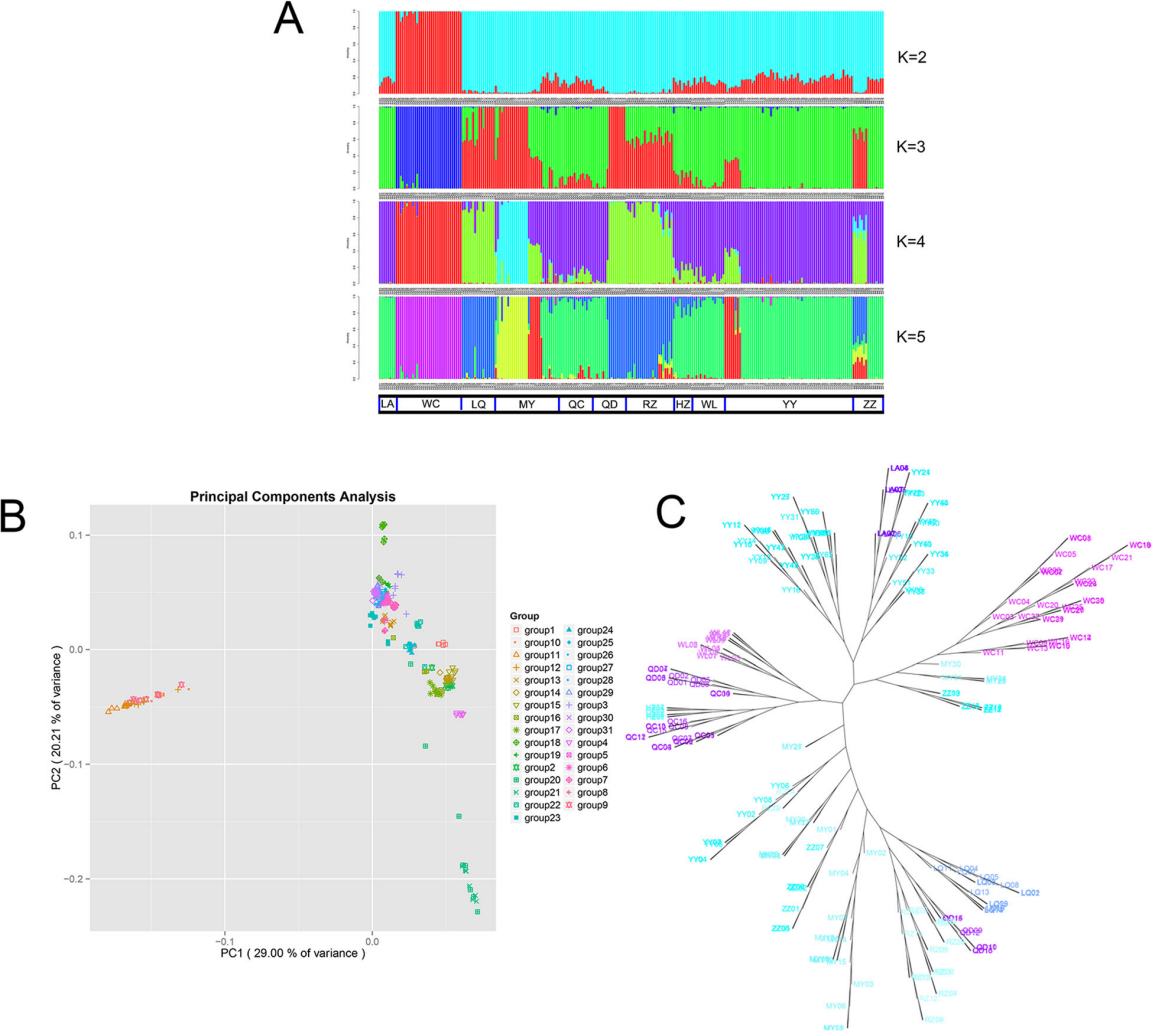
Population structure analysis results. **a** Stacked diagram generated using STRUCTURE. **b** Scatter plot of principal components 1 and 2 (PCA1 vs. PCA2) from 31 populations collected from 11 regions. Groups 1–31 correspond to LQ1, LQ2, QD1, QD2, WL1, WL2, QC1, QC2, WC1, WC2, WC3, WC4, HZ1, RZ1, RZ2, RZ3, ZZ1, ZZ2, LA, MY1, MY2, MY3, MY4, YY1, YY2, YY3, YY4, YY5, YY6, YY7, YY8 (**c**) Individual bee are adjacent to phylogenetic trees

We used STRUCTURE 2.3.4 for cluster analysis [26] (Fig. 4a). When the *K* value was 2, *A. cerana* in the WC region was divided into one class, and the other regions formed a large group with clear boundaries between regions. The results of the principal component analysis (PCA) scatter plot of principal factor 1 (variation 29%) and principal factor 2 (variation 20.21%) further supported the patterns (Fig. 4b) [27]. When the *K* value was 3 or 4, the large clusters were split. The central region (HZ, WL, QC) formed its unique pedigree composition with LA, and most of YY (YY1-YY7) formed a cluster. LQ and RZ formed a cluster; MY, ZZ, and QD showed different degrees of pedigree, and approximately one-half of the MY area (MY1 and MY2) showed a special lineage with clear differences in the area. When the *K* value was 5, the differentiation of MY, ZZ, and QD was further emphasized. MY was split into three areas, ZZ was split into two areas, and RZ was split into two areas, showing a highly mixed state. These results were further supported by the phylogenetic tree of the SNP data, as analyzed with Tessal 5.0 [28] (Fig. 4c). WC did not split as a whole, and most of YY and LA were close. A small region of YY was split with MY, ZZ, MY, and QD were highly split. HZ, WL, and QC were clustered into one class. LQ, RZ, and part of QD and ZZ were clustered into a large cluster, which is consistent with the results of STRUCTURE at *K* values ranging from 3 to 5. ΔK was determined by the Harvester method [29], where the best *K* value was 3, and all regions were divided into 4 genetic components; blue region (WC), green region (LA, YY, HZ, WL, QC), red region (LQ, RZ), and a mixed area of red and green (MY, QD, ZZ).

We studied the environmental characteristics of the bee sampling sites because they play important roles in the differentiation of *A. cerana* (Table 1). We divided the bees collected from the measurement areas into island type (WC), mountain type (LA, YY, HZ, WL, QC, MY), and plain type (QD, ZZ, LQ, RZ), which was consistent with the results of STRUCTURE. We found that climate can differentiate mountain-type bees, such as those from central and northern regions (Fig. 3c).

#### Genetic differentiation

Prior to genetic diversity analysis, we performed secondary filtering of the SNP data. SNP loci that could be classified in < 80% of individuals were excluded, and SNP loci with minor allele frequency (MAF) < 0.01 were excluded. Finally, only biallelic genes (−min-allele 2 - max-allele 2) and SNP loci with *P*-values < 0.01 according to the Hardy-Weinberg equilibrium test were filtered out. Finally, 2737 high-quality SNPs were used for the analysis (Additional file 12). The F-statistics (include *F*is, *F*st, and *F*it; Additional file 4: Table S3) of each SNP locus in all individuals were statistically analyzed using Genepop software (ver. 4.2.2) [26]. To balance the sample size, we selected a similar number of bees from each region. The paired *F*st between populations was calculated to quantify the genetic differentiation (Table 2). The *F*st ranged from 0.041 (HZ, QC) to 0.257 (AH and WC), with an overall mean of 0.132, indicating moderate genetic differentiation. The states (0.05 < 0.132 < 0.15) in which WC, LA, and LQ were highly differentiated (average *F*st, > 0.15) were consistent with the overall heterogeneity of the population structure and gene flow. Compared with *A. mellifera* (average *F*st, 0.10) [30], *A. cerana* was more highly differentiated at the level of genetic differentiation (average *F*st, 0.132). The *F*st difference between the different regions of the bee was large, showing rich species diversity at the ecotype level [30, 31]. *A. cerana* (SX, QC, WL) in the central mountainous region had the lowest *F*st distribution (average *F*st, 0.103), and WC had the highest differentiation state (average *F*st, 0.216). The region of North China (LA, YY, MY, QD, LQ, RZ, ZZ) showed variable differentiation states, with most being in a low differentiation state, although LA and LQ were in a highly differentiated state. We found that the mountainous regions (YY, HZ, WL, MY, QC) were higher than the other regions. Given the lower average *F*st of 0.166, we calculated the *F*st (Additional file 5: Table S4) and gene flow (Additional file 6: Table S5) among all groups. In the central mountainous region (HZ, WL, QC), the lowest Fst average value and the highest gene flow mean indicated that the genetic composition of the central mountainous region was similar, and the degree of differentiation was low, whereas *A. cerana* in the plain region showed a high degree of differentiation.

The polymorphism information content (*P*ic), effective allele number (*N*e), nucleic acid diversity (*P*i), heterozygosity (*H*o), and expected heterozygosity (*H*e) of each SNP locus in the population were separately calculated (Table 3). The number of effective alleles in the region was 1.46 (accounting for 73.05%). *P*ic measured the degree of variation of the population DNA (the overall *P*ic average was 0.236). Among these regions, the QC region (QC1, QC2) has the lowest *P*ic at 0.209, whereas the average *P*ic of the QD region (QD1, QD2) was the highest at 0.274. The more isolated populations had lower genetic polymorphisms (YY, HZ, WL, QC) with an average *P*ic of 0.214, which is consistent with the distribution of *P*i. Nucleotide diversity is an indicator of diversity within or between populations. The *P*i distribution of YY, HZ, WL, and QC was similar with average *P*i values of 0.281, 0.275, 0.253, and 0.266, respectively, which are lower than the average (the average *P*i = 0.306). Furthermore, the WC region surpassed the mean polymorphic information (*P*i = 0.321 > 0.306), and there was high differentiation (mean *F*st = 0.216) and low genetic variation (low polymorphism, *P*ic = 0.242 < 0.25). At the same time, *F*is < 0 indicated that the Wenchang area was in a closed island, and it had a large surface area, which caused *A. cerana* in the WC area to have more biodiversity and heterozygosity than other areas. There were significant differences among heterozygosity (*H*e), variance (*P*ic), and diversity (*P*i) (*P* < 0.01) in MY, among which MY2 and MY3 had higher heterozygosity, variation, and diversity, and MY1 and MY4 had lower heterozygosity, variation, and diversity. A comparison of the STRUCTURE stacking map with a K value of 3 (Fig. 4a) revealed that the MY area was divided into three categories, whereas MY1 and MY2 belonged to one category. MY3 was in the mixed area, belonged to the second category, and MY4 belonged to the third category with a slight inbreeding phenomenon (*F*is = 0.05 > 0). In addition, a large gene flow was observed between MY1 and MY2 (*N*m = 18.1). MY2 had higher heterozygosity (*H*e = 0.364), whereas MY1 remained relatively stable (*H*e = 0.28), suggesting that the genes flowed from MY1 to MY2. Overall, we found that *H*o was greater than *H*e at the population level and that the inbreeding coefficient was generally less than zero. We also calculated all sampled groups with an average observed heterozygosity (*H*o) of 0.3368, which was higher than the expected heterozygosity (*H*e) of 0.2865. The QD region (QD1, QD2) has the highest *H*o, *H*e and *P*ic (0.34, 0.43, and 0.274, respectively) indicated that the population heterozygosity was high, which could be affected by the combined effects of selection, mutation and genetic drift.

**Fig. 5:**
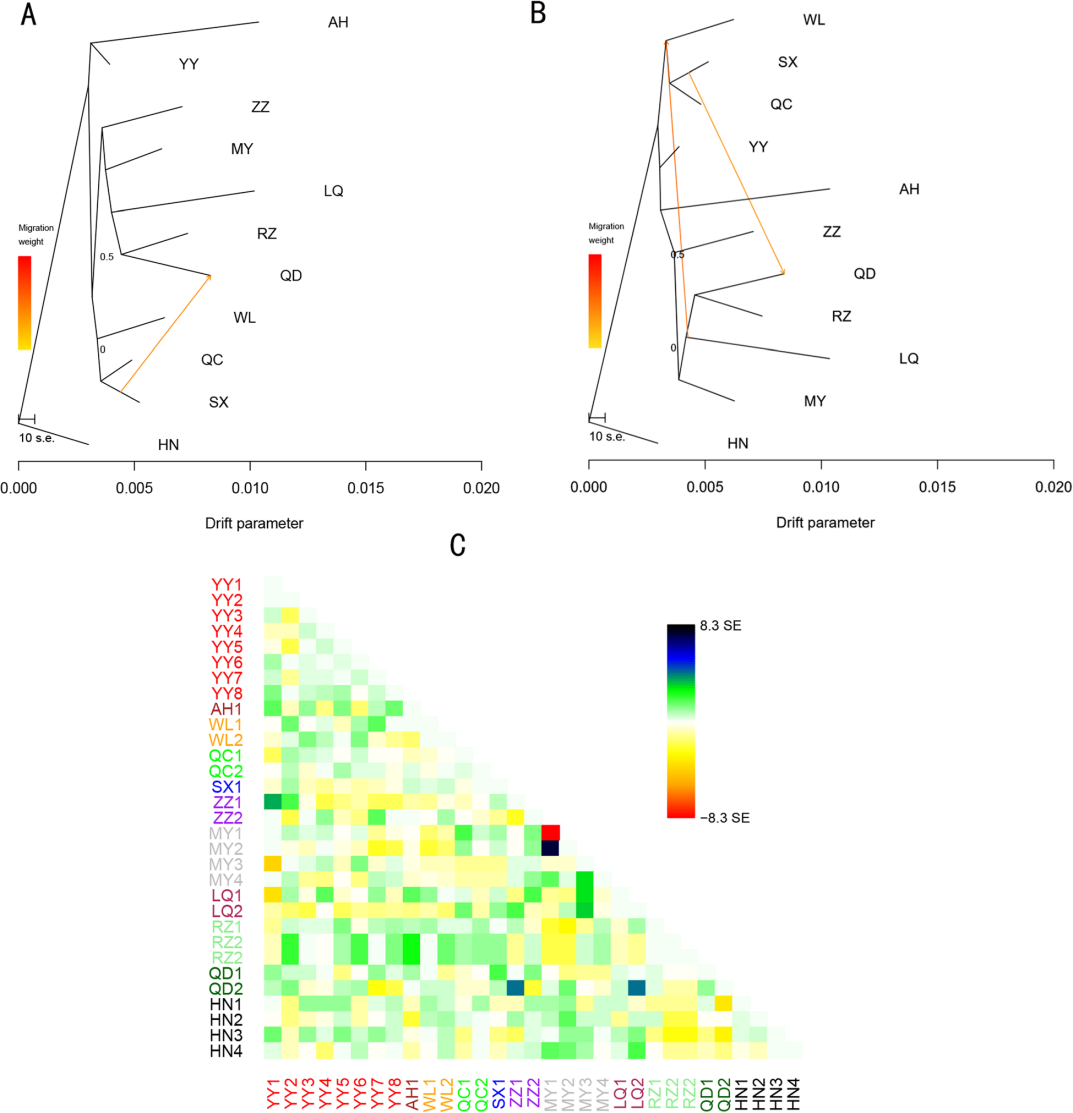
**a** Maximum likelihood tree with two migration event. The different regions are colored according to the geographical location, and the scale shows 10 times the average standard error of the sample covariance matrix W. **b** Maximum likelihood tree with two migration events. **c** Residual map of the residual fit of the maximum likelihood tree calculated using 31 populations without migration. We divided the mean standard error between all pairs of residual covariances *i* and *j* between each pair of populations. We then plotted the scaled residual. The color is described in the palette on the right. A positive residual value indicates that the groups are more closely related to each other

Treemix was used to construct the maximum likelihood tree with 1 and 2 migration events between regions in our study [32] using the graph with the highest probability of 100 independent runs in the species tree model (Fig. 5a, b). Modern populations shared common ancestors through shared branch points. The results of the tree model were consistent with the population state and phylogenetic tree (Fig. 4c) at a *K* value of 3 in the STRUCTURE stacking map (Fig. 4a, b), which largely demonstrated the known relationships between different groups. This result also explained the correlation between populations. The QD, RZ, and LQ regions of *A. cerana* evolved from MY and had a migratory relationship with the central region (WL, SX, QC). The first migration showed that the bloodline of QC was 18.1% for HZ (*w* = 18.1%). The second migration showed that the ancestors of HZ, QC, and WL were the ancestors of QD, RZ, and LQ (*w* = 26.5%). We evaluated the residual model using the 31 populations without migration to reveal the nonstrict hybrid events. All reported migration edges had *P*-values < 0.01. The three-population test was performed based on the “*f*3 structure” (Additional file 7: Table S6) [33] to further understand the characteristics of the mixed population. We calculated the corresponding *f*3 statistic for all possible combinations of the three mixed populations. The negative values indicated that there was mixing between the populations. The results indicated that only MY1 and MY2 showed a clear mixed signal. Compared to other regions, the WC region had the highest *f*3 statistic. We used all sampled groups information to generate a population maximum likelihood tree (Additional file 1: Figure S1), and the residual model was used to obtain a more intuitive understanding of the mixing between the populations (Fig. 5c) [32]. We found that MY and LQ, QD and ZZ, LQ had mixed events, which reveal to some extent the phenomenon of genetic structure divergence in the MY, QD and ZZ regions (Fig. 4a, *K* = 3–5).

#### Mantel test between *A. cerana* groups

The difference between the genetic distance and geographical distance calculated by the paired *F*st is one of the most common methods for assessing the spatial communication of the population structure [34–36]. We used ade4 within R [37], and the physical distance and genetic distance *F*st were tested by Mantel [34, 36]. We observed a significant correlation between geographical distance and genetic distance (*P* = 0.008). We also used PASSaGE for repeated tests and obtained the same result (*P* = 0.0093). There was a significant correlation between physical distance and genetic distance, and the correlation degree was *r* = 0.34493. We calculated this value for *A. cerana* in North China. The results showed that there was a weak correlation between geographic distance and genetic distance (*P* = 0.051), and the correlation degree was reduced to *r* = 0.273. This finding may be related to the high plains in North China and low altitude.

**Fig. 6:**
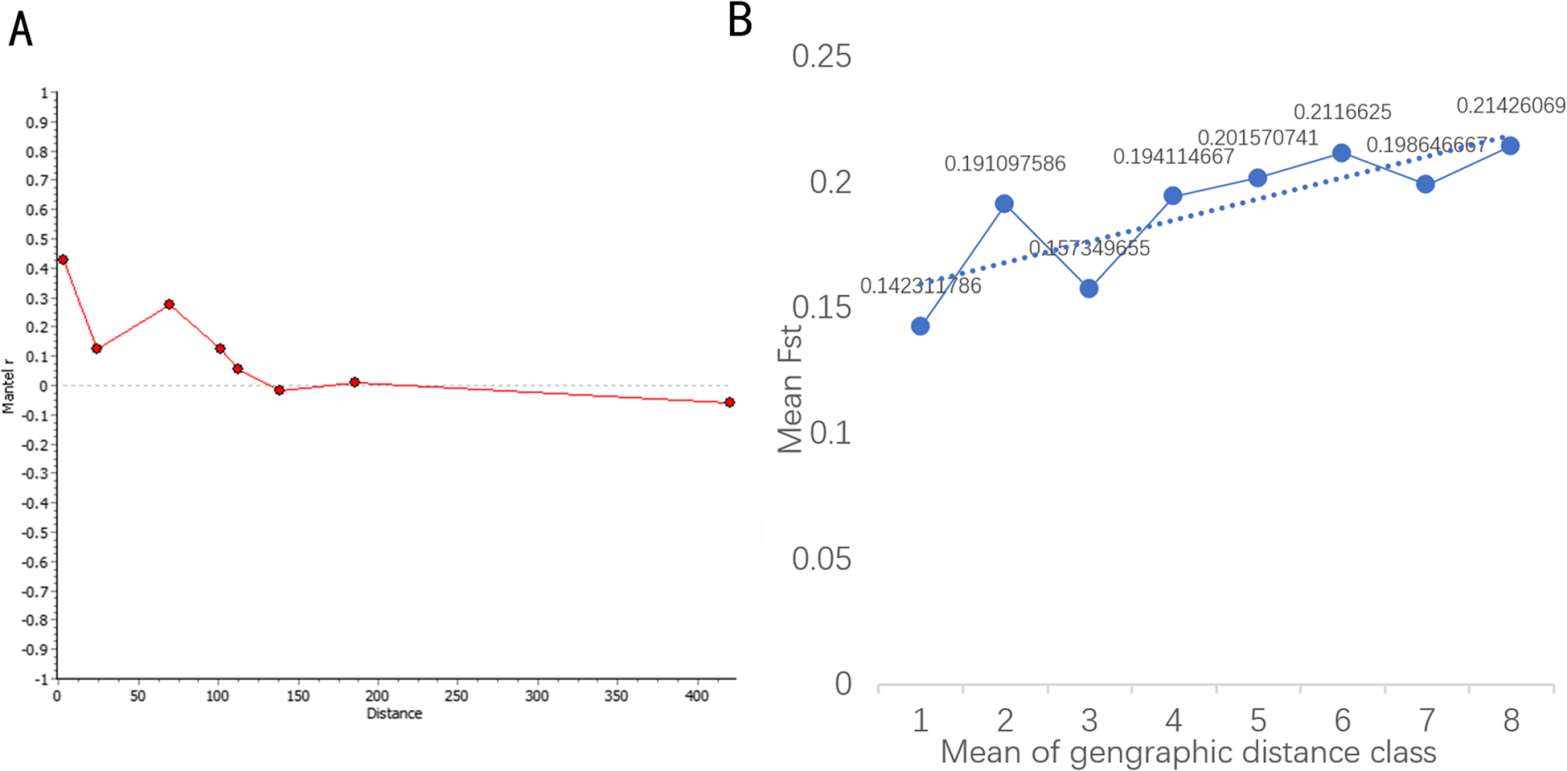
Manchester correlation diagram (**a**) and interference diagram (**b**), the latter by the average *F*st in each distance class

North China has a rich terrain and belongs to the temperate monsoon climate. To further understand the changes in the correlation between genetic distance and geographical distance, we separately calculated the *F*st and Manchester correlation of *A. cerana* in North China (Additional file 8: Table S7, Fig. 6a) [38, 39]. The most important parameter in the correlation diagram is that it should capture a continuous distribution within the geospatial space [40], and we divided *A. cerana* from North China into eight segments (Additional file 9: Table S8). When the observation sample was the same for each segment (0–6.23 *K*m), the population similarity was higher (*r* = 0.42; *P* < 0.01) in the first segment. When the distance was 156.4 *K*m, the population similarity decreased to 0 and then changed to a negative value, indicating that the population is not genetically similar. To make it easier to observe the Manchester correlation graph, we simultaneously calculated the *F*st value at each node and created an interference graph (Fig. 6b). The results show that the spatial structure is not strong, and the mixed terrain of the North China Plain and the mountain could be the cause of fluctuation. In general, when the geographical distance increases, the genetic similarity of *A. cerana* in North China fluctuates and shows a linear decreasing trend (lower correlation and higher *F*st value).

#### Candidate genes under natural selection

**Fig. 7:**
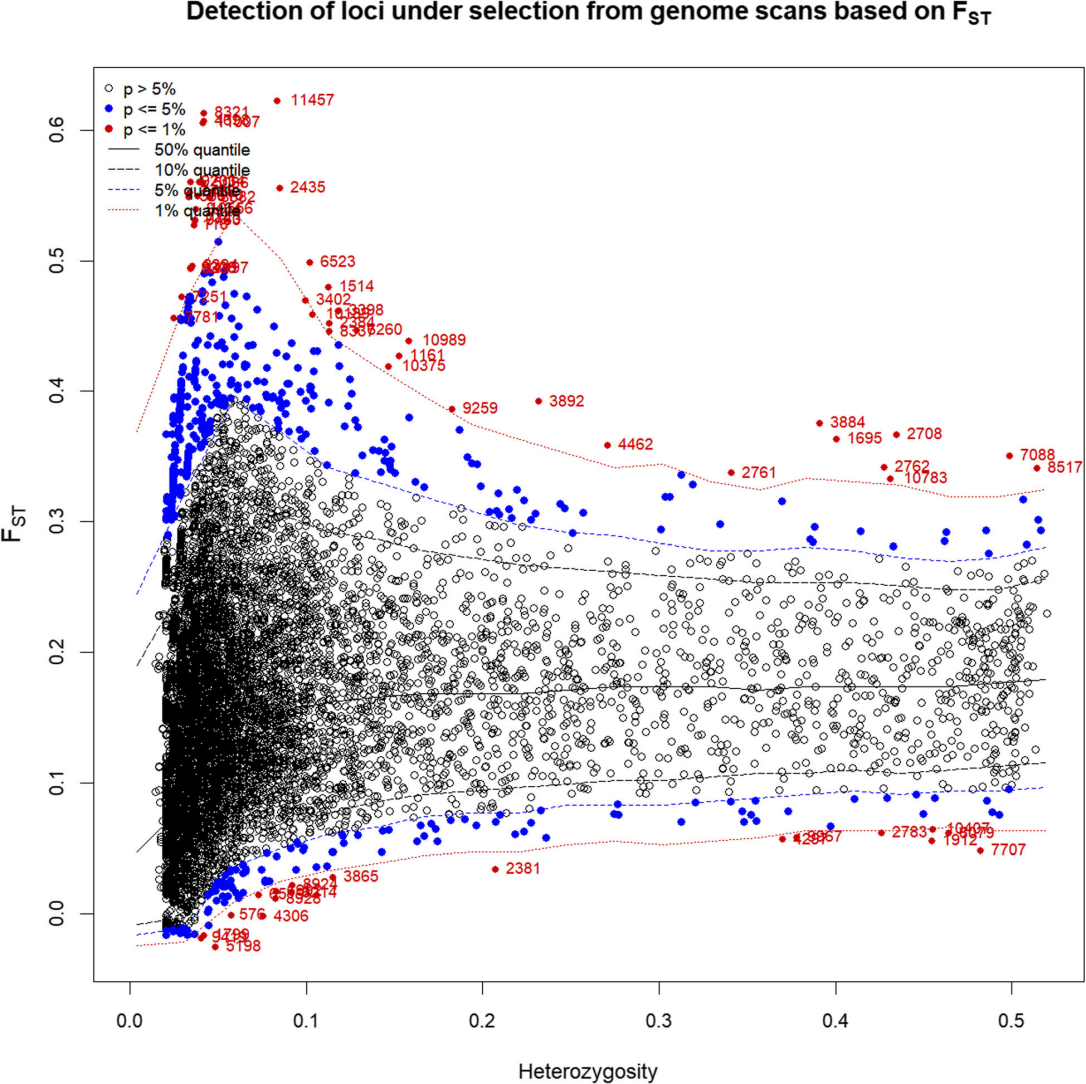
Identifies the different SNP loci assumed under the selection of the direction of the *F*st outlier method. Use The hierarchical model Arlequin 3.5. was used. *F*st: site-specific genetic differences between populations; heterozygosity: a measure of heterozygosity at each locus. Significant sites are indicated by red dots (*P* < 0.01)

We examined divergent SNP loci in 243 *A. cerana* according to the Arlequin stratification method and identified 121 candidate SNPs at a significance level of 1% (Fig. 7). A list of these SNP loci, together with the *A. cerana* genomic information obtained from the alignment with *A. mellifera* and *Drosophila* genomes, is shown in Additional file 10: Table 9.

According to Gene Ontology (GO) analysis and the related candidate genes in the Kyoto Encyclopedia of Genes and Genomes (KEGG) pathway, 20 significantly enriched GO terms and three significantly enriched KEGG pathways were selected (Table 4), in which the most significant was the Wnt signaling pathway. The Wnt pathway is highly conserved among different species, where it plays an important role in development, such as in the formation of *Drosophila* fins [41, 42]. In *A. mellifera*, the Hippo signaling pathway is generally considered to play an important role in cold climate adaptation [43], and a similar pathway has been identified in *A. cerana*, which is indicative of convergent evolution between the two species [19]. Metabolic pathways underlie how organisms respond to environmental stress, and studies have shown that parasitic infections, pesticides, and malnutrition can affect metabolic pathways in bees and reflect the adaptability of bees to changing environments [44].

**Fig. 8:**
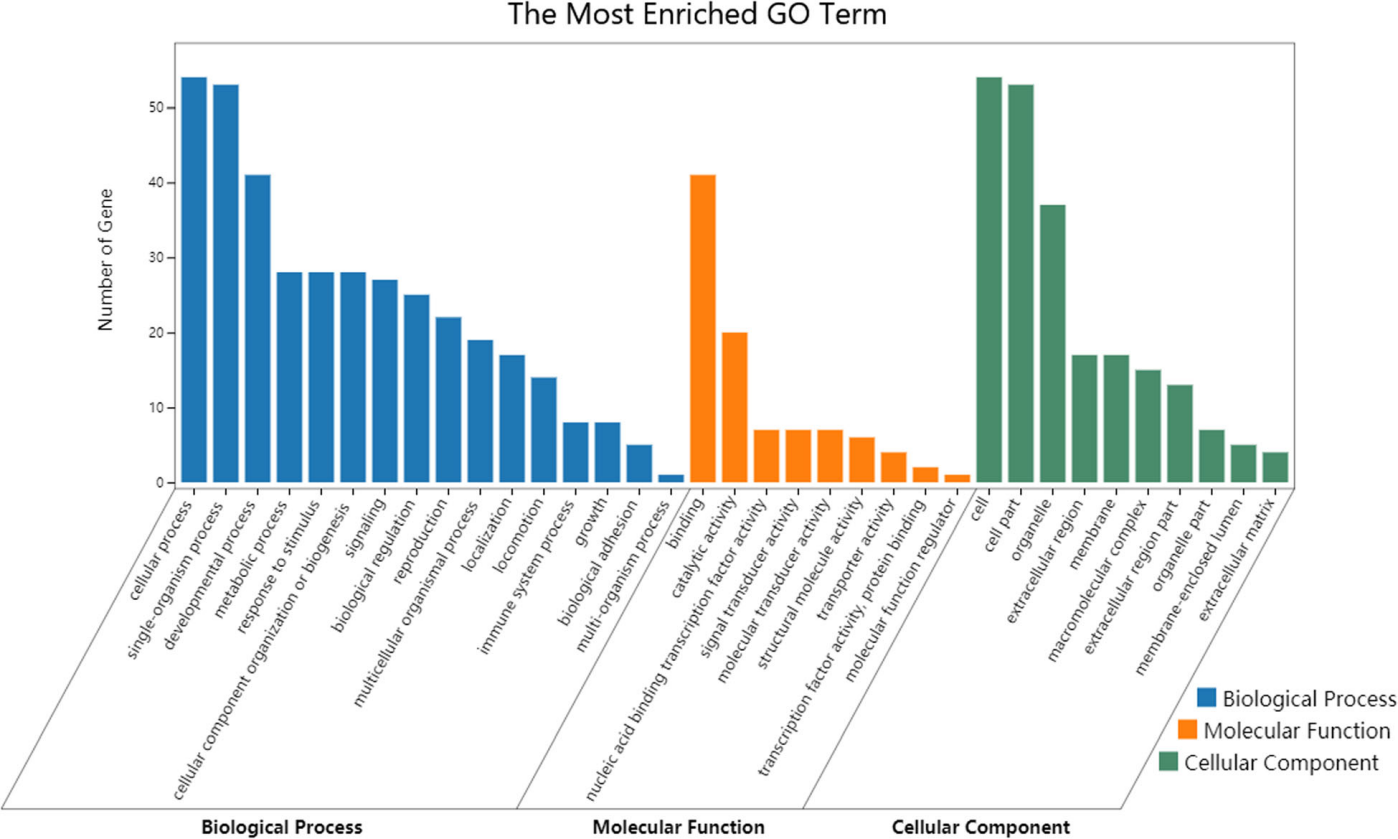
GO terms enriched by abnormal genes. The asterisks represent significant terms

For the enriched GO terms, adaptive terms, such as “response to stimulus”, “signal transduction”, “locomotion”, and “biological regulation”, were significant (Additional file 11: Table S10), indicating the importance of the processing of signals and responses when bees adapt to different environments. We also identified key enriched GO terms (Fig. 8), “developmental process”, “metabolic process”, “biological regulation”, “immune system process”, “catalytic activity”, and other genes related to growth and development that are involved in natural selection.

**Fig. 9:**
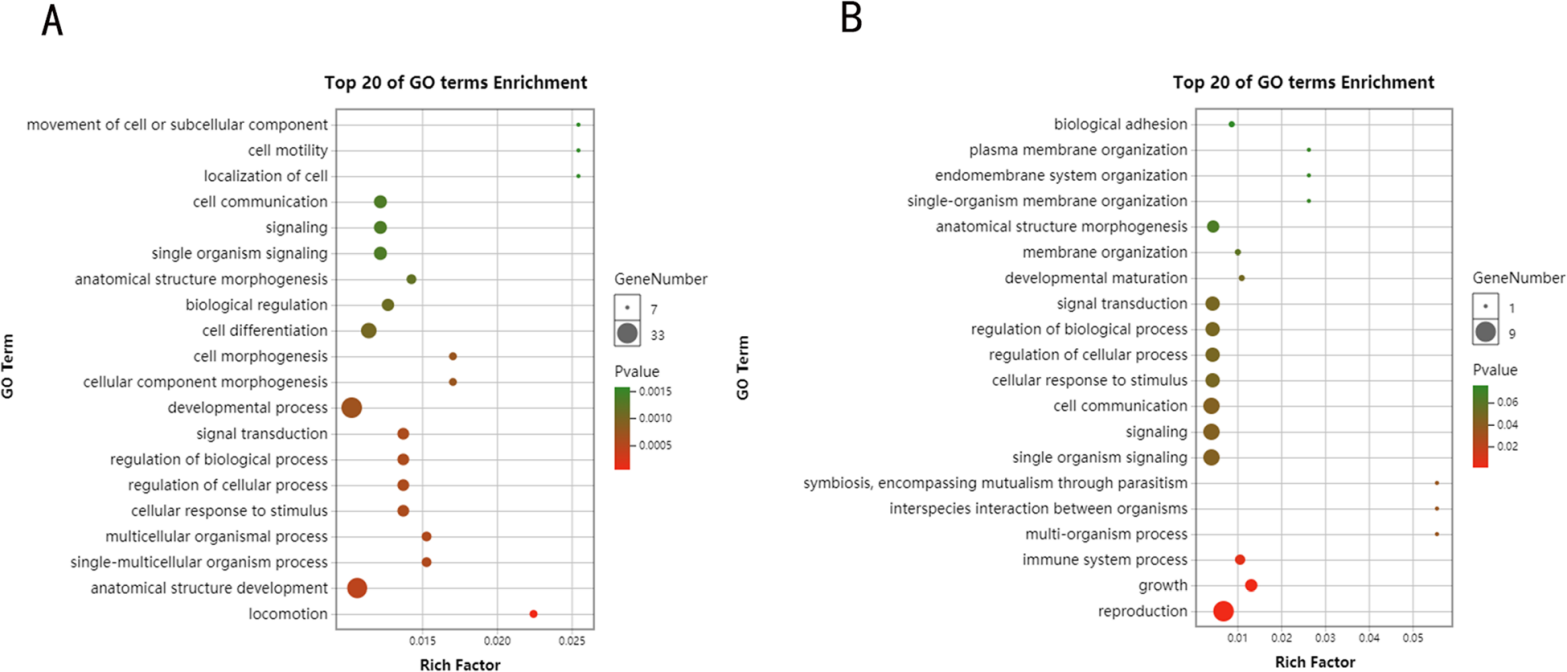
The left image shows the top 20 GO terms under directional selection, and the right image shows the top 20 GO terms under balanced selection. Rich Factor: Ratio of enriched gene number to total gene number in each GO term. Dot size were decided with enrichment gene number. Dot color were decided with enrichment *p*-value

We further analyzed the genes under balanced selection and directed selection. Among the genes corresponding to a total of 121 abnormal SNP loci, there were 30 genes under balanced selection and 91 genes under directed selection. We separately performed enrichment analysis of the genes under balanced selection and directed selection. Under balanced selection, the GO terms that were enriched in response to the stimuli, such as “locomotion”, “signal transduction”, and “biological regulation”, we found that directional selection under natural selection allowed the adaptive evolution of organisms (Fig. 9). However, the enriched GO terms “symbiosis, encompassing mutualism through parasitism”, “signal transduction”, “interspecies interaction between organisms”, and “information processing” indicated that balanced selection maintained the diversity of alleles between bees. Therefore, collaboration, communication, and other activities are important for the survival of bee populations. For instance, collaboration is critical for parasite identification, and foraging behaviors, highly diverse symbiosis, and genes involved in communication are conducive to interactions among bees and their ability to cope with changing environments.

## Discussion

The evolution of species and the generation of diversity are mostly due to environmental changes. To understand the relationships between genetic differentiation and the environment, this study collected *A. cerana* from 11 regions in China and analyzed the morphological measurements. To obtain as much information as possible about local *A. cerana*, we include as many collection points as possible in one collection area. The samples were mainly collected from frame beehives, semiartificial beehives, including tree barrel hives and wall hole hives, and natural beehives. The domestication process of *A. cerana* is rather slow, and domestic bees and wild bees often undergo mutual transformation [11]. To ensure the stability of *A. cerana* society, all frame of artificial and semiartificial beehives have to mimic their natural beehives. Therefore, in this study, we believe that different beehives have negligible effect on *A. cerana* behavior. We used 39 morphological indicators to classify *A. cerana* and classified the different morphological indicators, such as color, angle of right forewing, and body length, to obtain more comprehensive classification results. Overall, the classification of the angle of right forewing was consistent with the classification that included all traits. Francoy et al. used wing morphology to quickly identify similarities among Africanized bees [45]. We believe that the wing morphology can also be used to rapidly identify species.

The phenotypes of ZZ, RZ, QD, and LQ in North China were similar to those in the central region, which seems counterintuitive. We performed a genome-level analysis, and the STRUCTURE results showed that there was considerable genetic diversity in ZZ, RZ, QD, and MY. At *K* = 5, MY showed three different pedigree types. The two migrations of the Treemix population phylogenetic tree revealed signs of migration for QD, LQ, RZ and the central region, while the residual plot showed signs of mixing between ZZ and QD. Previous studies on Italian honeybees (*A. mellifera*) showed that human-mediated gene flow led to the management of honey bee colonies High genetic diversity [46, 47], and the MY region may be the result of human action.

Genetic variation is critical for the survival of bees in changing climates. For bees, high genetic diversity at the population level can increase the adaptability of colonies, as bee colonies with higher diversity exhibit a more stable ethnic balance [48, 49], are more productive [50, 51] and are more resistant to disease [52]. We calculated the *F*st value of population differentiation, which showed a large difference between WC and the inland region (average *F*st = 0.216). The *F*st value was previously calculated for *A. cerana* at the subspecies level between the more isolated populations (ecological type), for which the average *F*st differentiation was 0.162 in the range of 0.099 to 0.228 [19]. We calculated the *F*st between MY, YY, LA and WL, HZ, and QC between 0.6 and 0.9, but the morphological data show that it belongs to a different ecological type. Therefore, we believe that the *F*st range of *A. cerana* should be between 0.06 and 0.228, while the *F*st range for the *A. mellifera* subspecies of the same lineage was 0.05 to 0.15 [30]. Clearly, relative to *A. mellifera*, *A. cerana* has a richer genetic diversity.

Although ZZ, RZ, QD, and LQ may have undergone mixed population events, they are currently in a state of equilibrium. Furthermore, different mixed regions have gradually formed relatively independent ecological types. Previous studies have suggested that bees migrate rapidly in areas without strong physical barriers, which can promote gene flow and reduce genetic differentiation. The rapid spread of bees in Australia and the Americas can better illustrate the potential for the rapid migration of bees [53–55]. The results of the Mantel test showed that geographical distance had little effect on population differentiation, which is consistent with the results of studies using microsatellite markers [56, 57]; this finding allows us to focus our interest on other environmental variables. Previous studies have suggested that islands and mountains can form physical barriers that, in turn, cause group isolation. Therefore, the rapid migration of honeybees without a strong physical barrier can promote gene flow and reduce genetic differentiation. In addition, population growth can promote gene flow between populations [19], but our research shows that gene flow in the mountains is more pronounced than in plain areas (Additional file 6: Table S5). In China, various human activities, such as the loss of agricultural land and urbanization, led to the loss of habitat of *A. cerana*, especially in the vast plains of North China, which are particularly conducive to urban construction. However, complex terrains are more resistant to human activities, which could be the reason for the higher degree of differentiation of *A. cerana* in ZZ, RZ, QD, and LQ. We believe that the isolation of *A. cerana* by human urban agglomerations is more stringent than the physical isolation caused by mountains.

Researchers believe that the survival of a species through climate change, habitat loss, and ecosystem changes is due to their physiological tolerance limits and resilience, ecological characteristics (e.g., behavior, heat tolerance), and genetic diversity [58]. *A. cerana* shows the potential of using all three strategies. Research has described a method to identify the performance of bee populations based on outliers [59, 60]. In this study, we screened 121 SNP loci, and functional enrichment analysis highlighted several processes such as “neurology”, “biological regulation”, “behavior and growth”, and “interspecies interactions between organisms”, suggesting that genes associated with these loci may experience selective stress as the population spreads through various unwelcome habitats. Sensory transduction refers to the conversion of input stimuli into signals received by the brain. Specific changes in these genes may require adaptation to different food sources or other resources in the new environment. Bees exhibit a range of different behaviors in a social environment, and hundreds of genes are involved in the brain function and physiological behavior of bees [61]. The social behavior of bees helps the population maintain the homeostasis of the nest and counteract changes in the external environment [62, 63]. Furthermore, chemical signals coordinate the behavior and physiology of colony members, and changes in protein coding sequences may be related to the evolution of chemical communication systems found in bees [64]. Genetic diversity is an adaptive evolution of biological individuals to changing environments. The ability to adapt to changing environmental variables strongly depends on sufficient genetic variation, the indirect impact of population size on evolution, and the balance between environmental rates [65]. We further analyzed 91 loci under balanced selection and directed selection. The results of the enrichment analysis showed that equilibrium selection plays an important role in maintaining the diversity of alleles and increasing the stability of the population. Directed selection leads to the unilateral adaptive evolution of the population. Taken together, the results of our study indicated rich genetic diversity in *A. cerana*.

In summary, as a wide-range species, *A. cerana* shows good potential for climate change, but the effects of stress, such as pesticides, pollutants, pathogens, parasites, and limited flower resources caused by human activities, are posing a larger threat to *A. cerana* in China [66, 67]. In this study, we provided new phenotypic and genomic insights for further climate adaptation studies that can enhance our understanding of bee health and breeding management.

## Conclusions

Our study utilized the wide-range *A. cerana* species, as well as morphological characteristics and genomic methods, to provide insights into the differentiation and adaptation of the species in China. We proposed a method for the rapid identification of the morphology of the wing and found some historical migration between the North and Central regions. *A. cerana* in China exhibits high genetic diversity, and physical barriers, rather than distance, are the driving factors for the differentiation of this highly migratory species. Human activities, such as urbanization, have a great impact on the differentiation of *A. cerana*. We screened for abnormal SNP loci and obtained related genes, performed enrichment analysis and gene ontology categories to identify candidate genes. The results of this study may help to elucidate the evolution of *A. cerana* in different environments and promote our understanding of how bee populations will respond to increasing climate change and how to protect bees from current and future challenges.

## Methods

### Sample collection

**Fig. 10:**
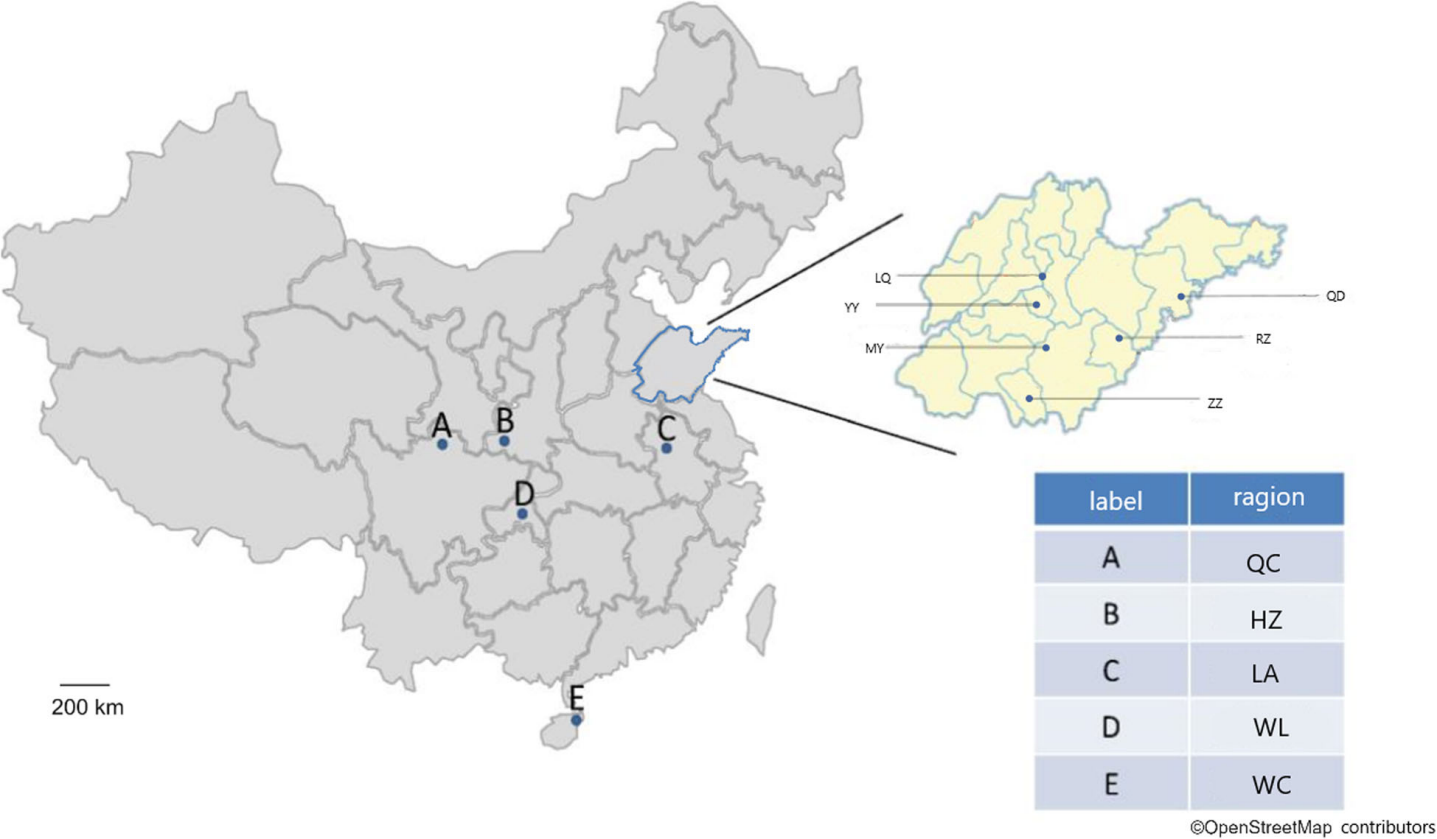
The position of the sample in China, where the blue area represents the dense sampling site of the representative area of North China f10:2 (Shandong). Sampling positions in Shandong Province were specially highlighted as yellow magnified area map. Base layer map date©OpenStreetMap contributors (page: https://www.openstreetmap.org/copyright)

Samples were collected from six regions, Mengyin (MY), Yiyuan (YY), Rizhao (RZ), Qingdao (QD), Linqu (LQ) and Zaozhuang (ZZ), in Shandong (SD) Province and *A. cerana* germplasm conservation areas or traditional breeding sites in 11 provinces and municipalities, such as Chongqing (WL), Sichuan (QC), Anhui (LA), Shanxi (HZ) and Hainan (WC) (Fig. 10). To obtain as much information as possible about local *A. cerana*, we include as many collection points as possible in one collection area. The samples were mainly collected from frame beehives, semiartificial beehives, including tree barrel hives and wall hole hives, and natural beehives (Table 5). All frame beehives and semiartificial beehives are designed to mimic natural beehives. At least one sample group of ~ 30–50 adult worker bees at each sampling point, after being soaked in absolute ethanol and placed in a sealed grinding bottle, were brought back and stored in a − 20 °C freezer.

**Table 1:** Type of climate between different regions

Region	Altitude	Climate type
MY	314.675	Temperate monsoon climate
YY	309.875	Temperate monsoon climate
RZ	148.8	Temperate monsoon climate
QD	137.9	Temperate monsoon climate
ZZ	221.75	Temperate monsoon climate
LQ	168	Temperate monsoon climate
WL	1221	Subtropical monsoon climate
QC	908	Subtropical monsoon climate
LA	337	Temperate monsoon climate
HZ	1260	Subtropical monsoon climate
WC	28.475	Tropical monsson climate

### Dissection and collection of morphometric data

This test was carried out in accordance with the indicator project of the honeybee morphology researcher [68, 69] and the color standards of *A. cerana* [70]. First, a picture of the entire body, which included the color pattern of the bee specimen, was taken, and tweezers were used to remove the front and rear wings from the chest. The sternal remnants were removed by tearing the connective tissue between the wings. The cut sternum was cleaned with a soft-bristle brush, and the residual tissue was removed and fixed to the assay plate with scotch tape. Using a specific measuring microscope and its matching measurement software ImageView 3.7, morphological measurements were performed after adjusting the scale. The morphological markers were as follows: front and rear wing length; angle of right forewing (A4, B4, D7, E9, K19, G18, J10, L13, J16, N23, O26); elbow pulse length; and color aspect measurement of the lip base, upper lip, small scutellum SC area, K area, B area, second back board, third back board, and fourth back board (a total of 39 index). To avoid bias and improve accuracy, each sample was evaluated for pigmentation by three different observers, and the highest number of each score was recorded and used for subsequent analysis.

### Sequencing, read mapping, and quality control

*A. cerana* was randomly selected from each of the sampled sites for sequencing, and a tag sequencing library of *A. cerana* samples was constructed by the 2b-RAD technique. All samples were constructed using standard 5′-NNN-3′ linkers. To obtain high quality reads, both the library and quality-controlled libraries were subjected to paired-end sequencing data filtering on the HiSeq X Ten platform, and SOAP2 was used to compare the sequencing data to the genome [71, 72] using maximum likelihood (maximum likelihood, ML) perform site typing and SNP detection, as well as VCF tools, to carry out strict filtering to obtain the most informative SNPs [60]. The detected SNPs were filtered out as follows: (1) except for sites in which less than 80% of the individuals could be typed, (2) sites with an MAF below 0.05, (3) one or four allelic sites were excluded, (4) exclude more than one SNP locus in the tag, (5) eliminate loci with a genotype deletion ratio greater than 0.2, (7) exclude sites with a sample deletion ratio of 0.2 or higher, and (8) filter out the Hardy-Weinberg equilibrium test for *p*-values less than 0.05. A total of 11,504 SNP loci were obtained for all samples (Table 6).

**Table 2:** Paired *Fst* genetic distances between *A. cerana* populations

Pop.	LA	WC	LQ	MY	QC	QD	RZ	HZ	WL	YY	ZZ
WC	0.257										
LQ	0.204	0.252									
MY	0.139	0.194	0.127								
QC	0.126	0.185	0.128	0.061							
QD	0.167	0.227	0.143	0.103	0.093						
RZ	0.178	0.245	0.156	0.115	0.11	0.122					
HZ	0.139	0.192	0.131	0.069	0.041	0.093	0.118				
WL	0.147	0.198	0.142	0.076	0.058	0.107	0.126	0.062			
YY	0.149	0.202	0.142	0.078	0.064	0.105	0.121	0.075	0.081		
ZZ	0.15	0.204	0.141	0.081	0.079	0.11	0.12	0.083	0.092	0.087	
Ave	0.166	0.216	0.157	0.104	0.095	0.127	0.141	0.1	0.109	0.11	0.115

**Table 3:** Genetic diversity information of 31 *A. cerana* groups

Group	Ne	He	Ho	PIC	Fis	Pi
YY1	1.512	0.309	0.388	0.251	−0.253	0.331
YY2	1.385	0.248	0.284	0.207	−0.112	0.264
YY3	1.397	0.257	0.288	0.215	−0.133	0.274
YY4	1.396	0.255	0.289	0.213	−0.114	0.272
YY5	1.417	0.27	0.301	0.226	−0.017	0.294
YY6	1.394	0.254	0.29	0.213	−0.033	0.271
YY7	1.415	0.264	0.31	0.219	−0.055	0.282
YY8	1.387	0.251	0.28	0.21	−0.1	0.267
LA	1.523	0.319	0.379	0.26	−0.198	0.341
WL1	1.408	0.258	0.298	0.214	−0.113	0.275
WL2	1.407	0.258	0.296	0.214	−0.092	0.275
HZ	1.356	0.237	0.246	0.201	0.004	0.253
ZZ1	1.46	0.286	0.333	0.236	−0.136	0.308
ZZ2	1.543	0.324	0.418	0.261	−0.243	0.346
MY1	1.449	0.28	0.317	0.231	−0.131	0.3
MY2	1.611	0.364	0.432	0.293	−0.196	0.389
MY3	1.569	0.333	0.446	0.268	−0.19	0.36
MY4	1.345	0.232	0.239	0.197	0.005	0.247
LQ1	1.545	0.322	0.413	0.26	−0.239	0.345
LQ2	1.541	0.325	0.393	0.263	−0.158	0.347
RZ1	1.47	0.292	0.358	0.24	−0.178	0.311
RZ2	1.505	0.308	0.365	0.251	−0.129	0.328
RZ3	1.402	0.261	0.268	0.218	0.005	0.278
QD1	1.518	0.316	0.396	0.257	−0.213	0.337
QD2	1.613	0.362	0.459	0.291	−0.224	0.386
WC1	1.452	0.281	0.333	0.231	−0.127	0.3
WC2	1.509	0.308	0.37	0.251	−0.172	0.329
WC3	1.484	0.295	0.346	0.241	−0.145	0.315
WC4	1.519	0.316	0.367	0.257	−0.139	0.338
QC1	1.425	0.27	0.307	0.224	−0.085	0.288
QC2	1.338	0.228	0.233	0.194	0.015	0.243
Average	1.461	0.287	0.337	0.236	N/A	0.306

**Table 4:** Significantly enriched KEGG pathways

Category	Term	count	*P*-Value	Benjamini	Fisher Exact
KEGG_PATHWAY	Wnt signaling pathway	4	1.90E-02	3.30E-01	2.80E-03
KEGG_PATHWAY	Hippo signaling pathway-fly	3	5.40E-02	4.40E-01	6.90E-03
KEGG_PATHWAY	Metabolic pathways	5	8.90E-02	5.20E-01	8.00E-02

**Table 5:** Geographical information of the *A. cerana* sampling location

Province	Region	Longitude	Latitude	Nest/Hive type	Number
Shandong	MY	117.4954~118.0072	35.3608~35.5471	natural beehive	60
	YY	118.2056~118.3009	36.0034~36.0501	frame beehive	120
	RZ	119.2493~119.3662	35.6367~35.6802	frame beehive	45
	QD	119.7948~120.5137	35.8845~36.2050	frame beehive	30
	ZZ	117.6521~117.6927	35.0355~35.0431	natural beehives	30
	LQ	118.3106	36.1846	frame beehive	30
Chongqing	WL	107.7061	29.3941	semi-artificial hive	30
Sichuan	QC	105.1457	32.3455	semi-artificial hive	30
Anhui	LA	117.0127	31.408	frame beehive	15
Shanxi	HZ	116.4401	33.156	semi-artificial hive	15
Hainan	WC	110.7254~110.7805	19.5541~19.6845	semi-artificial hive	60

**Table 6:** Number of *A.cerana* SNPs detected in each functional area

SNP	Number
3_prime_UTR_variant	154
5_prime_UTR_premature_start_codon_gain_variant	29
5_prime_UTR_variant	167
downstream_gene_variant	606
intergenic_region	3048
intragenic_variant	24
intron_variant	5181
missense_variant	223
non_coding_exon_variant	5
splice_acceptor_variant&intron_variant	1
splice_donor_variant&intron_variant	1
splice_region_variant	4
splice_region_variant&intron_variant	23
splice_region_variant&synonymous_variant	10
start_lost	1
stop_gained	2
synonymous_variant	872
upstream_gene_variant	1153
total	11,504

### Morphometric analysis

We used SPSS 22 to analyze the morphological data of 462 *A. cerana*. First, we calculated the mean and coefficient of variation between the regions. ANOVA was used to determine which variables best distinguish the differences between the different regions, and principal component analysis (PCA) was performed. Then, we used the first two main factors of PCA (cumulative variability reached 57.4) and constructed different scatter plots. Finally, we use the hierarchical cluster function in SPSS 22, select Ward’s method to perform hierarchical clustering, and combine the most similar samples according to the degree of closeness between the different indicators. The sequential aggregation method classified all samples to obtain an intuitive understanding of the population structure.

### Population genetics analyses

Prior to population genetic analysis, VCF tools were used to rigorously obtain the most informative SNPs [60]. The inbreeding coefficient (*F*), expected heterozygosity (*H*e), and observed heterozygosity (*H*o) were calculated using PLINK2 [73]. The ratio of the minor allele frequency (MAF) to the polymorphic SNP (*P*ic) was calculated using a custom Perl script. The *F*is, *F*st, and *F*it values of the SNP locus in 243 samples were statistically analyzed using Genepop software (version 4.2.2) [26]. The Mantel test of the *F*st matrix and distance matrix was performed using the ade4 package in R [37]. We used TreeMix [32] to infer the history of the population division and mixture, allowing for three mixed events [33]. The method constructed a differentiation tree of the population and then identified the potential events of the population mixture from the residual covariance matrix.

### Bayesian population structure and principal component analysis

A model-based Bayesian clustering method was used to characterize the different genetic clustering patterns between the different regions with STRUCTURE 2.3.4 [74]. The genome of each bee was located in a predetermined set *K*, and the variable ancestor ratio was determined by the allele frequency of the population. This approach allowed the use of mixed bees to characterize ancestral populations [74]. We used a hybrid model and applied a putative number of clusters *K* from one to ten for 11,506 SNPs to run on STRUCTURE. The analysis was performed without prior knowledge of the demographic identity by simulating 50,000 preburning steps and 100,000 iterations of the MCMC algorithm per run. Ten independent runs were performed for each *K* to estimate the most reliable different genetic clusters using the probability of posterior probability (LnP(N/K)) [75] and the temporary amount DK of each *K* partition. The posterior probability variation with respect to *K* between the different runs was designated as the method for determining the true *K* value [76]. The most likely value for *K* was based on the network software STRUCTURE HARVESTER [29] and Evanno’s ΔK method [76], which was based on the average log likelihood, Ln P(D). Using TASSEL v5.0 [28] and SPSS 22 to perform *PCA* of the genetic and morphological traits of different individuals, respectively, enabled us to visualize the correlations between individual bees in individuals/regions on a multidimensional scale.

### Correlations between environmental variables and genetic diversity

The Mantel test was performed using the *F*st matrix and distance matrix with the ade4 software package in R [37]. To describe the possible changes in the correlation between the genetic distance and geographical distance, we used PASSaGE 2.0 for Manchester correlation analysis [77], according to the distance. The rank divided the distance matrix into submatrices. The populations within the boundary geographical distance described by each submatrix corresponded to populations with different genetic distances. The average *F*st of the set “distance level” was calculated separately to generate a polyline “interference graph” that was combined with the Manchester correlation map for a more intuitive view.

### Detecting SNP loci under selection based on *F*st outlier tests

A coalescence-based simulation was used to detect deviating SNP sites. With these methods, we expected to detect low-level differentiation sites under balanced selection (neutral loci) and high-level differentiation sites under directed selection (differentiation loci). The layering method we used was a modification of the FDIST method that was performed with Arlequin ver 3.5.2.2 [78, 79].. We used a hierarchical island model with 50,000 simulations to calculate the relationship between the *F*st and heterozygosity. A locus with an *F*st value higher than the 0.99 limit of the neutral distribution was considered to be a putative outlier under divergent selection [59]. The remaining loci with nonsignificant *F*st values were considered to be neutral SNPs. All procedures reduced bias and maintained highly differentiated loci between ecotypes individuals. We chose *F*st values that were higher than the expected neutral distribution as the directional selection sites and *F*st values that were lower than the expected neutral distribution as the balanced selection sites [60].

### Gene ontology analysis

SNP loci were screened using high-quality SNP loci [59, 80], and the reference sequence was obtained from NCBI (https://www.ncbi.nlm.nih.gov/assembly/GCF_001442555.1). A 50-base sequence upstream and downstream of the extraction site was sequenced and aligned with the *A. mellifera* sequence to obtain the most recent genes. Enrichment analysis was based on their orthologs in *Drosophila melanogaster*. The genes were assigned to GO and KEGG pathways. The functional enrichment of gene IDs was performed using GeneMania and g: Profiler [81, 82] with the online analysis site DAVID for KEGG pathway exploration [83] and Benjamini to correct the *P*-values to identify clusters of significantly enriched terms.

## Supplementary information

**Additional file 1: Figure S1.** Maximum likelihood tree with one migration event.

**Additional file 2: Table S1.** The 39 morphological measurements of *A. cerana*.

**Additional file 3: Table S2.** Abbreviation table.

**Additional file 4: Table S3.** The *F*is, *F*st, and *F*it of each SNP locus.

**Additional file 5: Table S4.** The *F*st table between all groups.

**Additional file 6: Table S5.** The gene flow table between all group.

**Additional file 7: Table S6.** Three population tests for admixture.

**Additional file 8: Table S7.** The Fst value table and distance table in North China.

**Additional file 9: Table S8.** Mantel test table.

**Additional file 10: Table S9.** Details of 121 candidate SNP loci.

**Additional file 11: Table S10.** GO term enrichment result.

**Additional file 12.** SNP data after filtering.

## Data Availability

The raw genome 2b-RAD tag-sequencing datasets generated during the current study are publicly available under NCBI Bioproject: PRJNA579872. All other data analysed in the present study are included in this published article (and its Supplementary Information files).

## Abbreviations

a: Length of bee right forewing cubital vein a
*A. cerana*: *Apis cerana*
*A. mellifera*: *Apis mellifera*
A4: Angle A4 of bee forwing
B: Bee pigment of scutellum B area
b: Length of bee right forewing cubita lvein b
B4: Angle B4 of bee forwing
Ci: Bee index number of cubital veins
D7: Angle D7 of bee forwing
E9: Angle E9 of bee forwing
FB: Width of bee right forewing
Fe: Length of femur of bee hindleg
FL: Length of bee right forewing
Fst: F-statistics, Genetic differences among population.
G18: Angle G18 of bee forwing
GO: Gene ontology
He: Expected heterozygosity
Ho: Heterozygosity
HZ: Shanxi
J10: Angle J10 of bee forwing
J16: Angle J16 of bee forwing
K: Bee pigment of scutellum Karea
K19: Angle K19 of bee forwing
KEGG: Kyoto Encyclopedia of Genes and Genomes
L13: Angle L13 of bee forwing
L7: Bee length of sternum 7
LA: Anhui
LQ: Linqu
MAF: Minor allele frequency.
ML: Length of bee hind leg metabasitarsus
MT: Width of bee hind leg metabasitarsus
MY: Mingyin
N23: Angle N23 of bee forwing
Ne: Effective allele number
Nh: Number of bee hindwing hamuli
NT: Bee branches midrib
O26: Angle O26 of bee forwing
P2: Bee pigment of tergum 2
P3: Bee pigment of tergum 3
P4: Bee pigment of tergum 4
Pc: Bee pigment of clypeus
PCA: Principal component analysis
Pi: Nucleic acid diversity
PIC: Polymorphism information content
PL: Bee pigment of labrum
QC: Sichuan
QD: Qingdao
RZ: Rizhao
S4: Bee length ofsternum 4
Sc: Bee pigment of scutellum Sc area
SNP: Single nucleotide polymorphism.
T3: Bee length of tergum 3
T4: Bee length of tergum 4
T5: Bee width of tomentum 1 ontergum 5
T7: Bee width of sternum 7
Ti: Length of tibia of bee hind leg
WC: Hainan
WD: Bee distance of bee wax mirror on sternum 4
WL: Bee length of bee wax mirror on sternum 4
WL: Chongqing
WT: Bee width of bee wax mirror on sternum 4
YY: Yiyuan
ZZ: Zaozhuang
