# Supplementary figures and images for: Population genomics and morphological features underlying the adaptive evolution of the eastern honey bee (Apis cerana)
Source: BMC Genomics. 2019 Nov 15;20:869. doi: 10.1186/s12864-019-6246-4 (PMC6858728; doi:10.1186/s12864-019-6246-4)

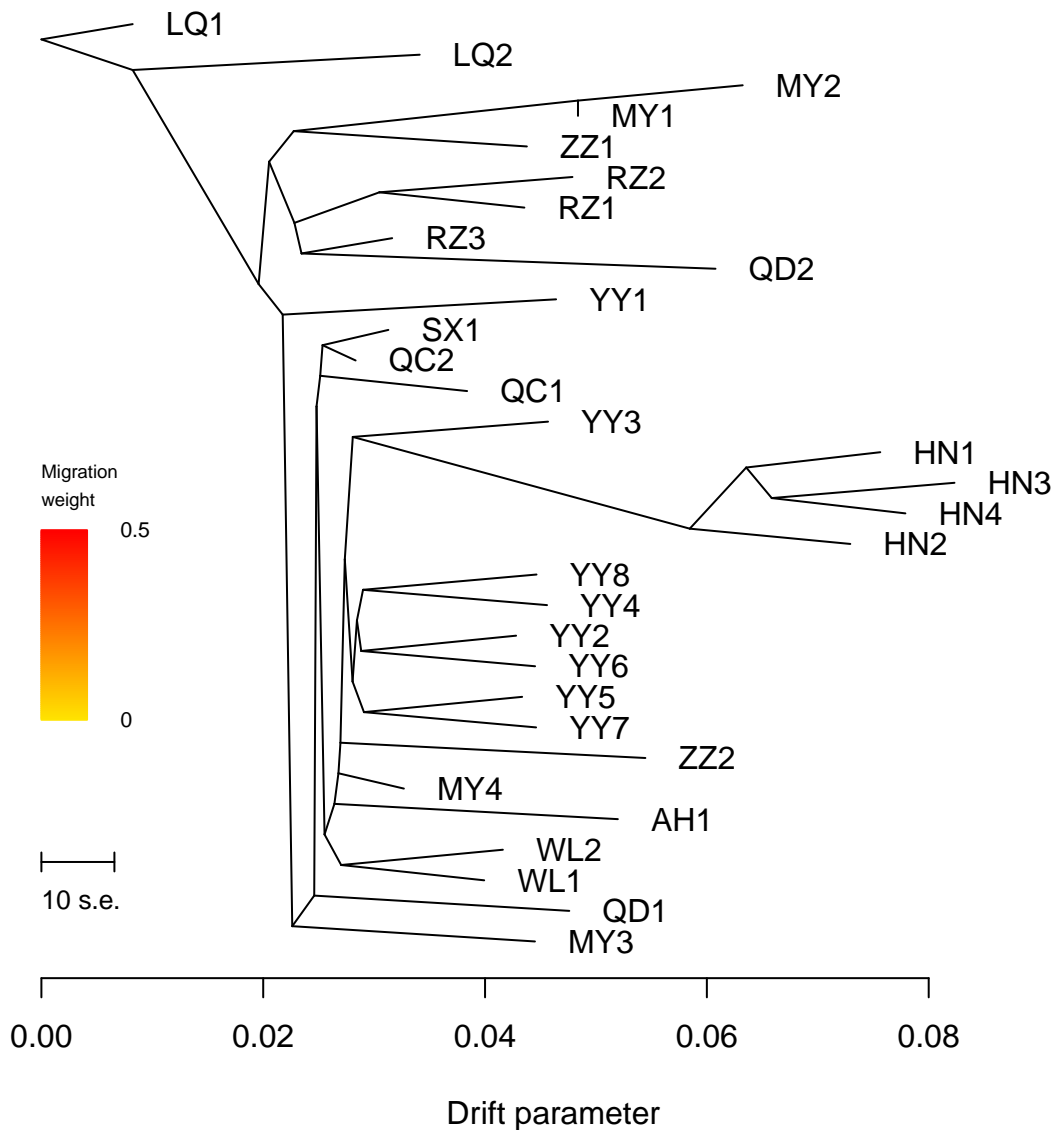

Supplement: Supplementary file 1 — Additional file 1: Figure S1. Maximum likelihood tree with one migration event. [file 12864_2019_6246_MOESM1_ESM.pdf]
